# Multivariate Analysis in Microbiome Description: Correlation of Human Gut Protein Degraders, Metabolites, and Predicted Metabolic Functions

**DOI:** 10.3389/fmicb.2021.723479

**Published:** 2021-09-17

**Authors:** Stefano Raimondi, Rosalba Calvini, Francesco Candeliere, Alan Leonardi, Alessandro Ulrici, Maddalena Rossi, Alberto Amaretti

**Affiliations:** ^1^Department of Life Sciences, University of Modena and Reggio Emilia, Modena, Italy; ^2^BIOGEST-SITEIA, University of Modena and Reggio Emilia, Modena, Italy

**Keywords:** gut microbiota, metagenomics, function prediction, protein fermentation, data fusion, ASCA

## Abstract

Protein catabolism by intestinal bacteria is infamous for releasing many harmful compounds, negatively affecting the health status, both locally and systemically. In a previous study, we enriched in protein degraders the fecal microbiota of five subjects, utilizing a medium containing protein and peptides as sole fermentable substrates and we monitored their evolution by 16S rRNA gene profiling. In the present study, we fused the microbiome data and the data obtained by the analysis of the volatile organic compounds (VOCs) in the headspace of the cultures. Then, we utilized ANOVA simultaneous component analysis (ASCA) to establish a relationship between metabolites and bacteria. In particular, ASCA allowed to separately assess the effect of subject, time, inoculum concentration, and their binary interactions on both microbiome and volatilome data. All the ASCA submodels pointed out a consistent association between indole and *Escherichia–Shigella*, and the relationship of butyric, 3-methyl butanoic, and benzenepropanoic acids with some bacterial taxa that were major determinants of cultures at 6 h, such as Lachnoclostridiaceae (*Lachnoclostridium*), Clostridiaceae (*Clostridium sensu stricto*), and Sutterellaceae (*Sutterella* and *Parasutterella*). The metagenome reconstruction with PICRUSt2 and its functional annotation indicated that enrichment in a protein-based medium affected the richness and diversity of functional profiles, in the face of a decrease of richness and evenness of the microbial community. Linear discriminant analysis (LDA) effect size indicated a positive differential abundance (*p* < 0.05) for the modules of amino acid catabolism that may be at the basis of the changes of VOC profile. In particular, predicted genes encoding functions belonging to the superpathways of ornithine, arginine, and putrescine transformation to GABA and eventually to succinyl-CoA, of methionine degradation, and various routes of breakdown of aromatic compounds yielding succinyl-CoA or acetyl-CoA became significantly more abundant in the metagenome of the bacterial community.

## Introduction

Proteins and peptides that escape digestion and absorption in the small intestine reach the colon, where they serve as a substrate for the resident microbiota. Protein breakdown in the colon is carried out by both human and bacterial proteases and peptidases, which provide the intestinal bacteria with building blocks for the anabolic processes. Nonetheless, the released peptides and amino acids fuel the catabolism of putrefactive bacterial groups gaining energy from the fermentation of amino acids.

While the intestinal fermentation of undigested carbohydrates mainly yields short-chain fatty acids (SCFA), which are generally recognized to positively impact the host’s health, the fermentation of amino acids yields a plethora of products, including some metabolites that are undisputedly detrimental to health (Yao et al., 2016; Kaur et al., 2017). The range of fermentation products generated by amino acid-fermenting bacteria encompasses ammonia, amines, and other nitrogen-containing compounds; linear and branched organic acids (acetate, butyrate, propionate, valerate, isobutyrate, 2-methylbutyrate, isovalerate, etc.); a variety of phenols and indole derivatives originating from aromatic amino acids; and sulfides originating from sulfur-containing amino acids (Fan et al., 2015; Oliphant and Allen-Vercoe, 2019). The detrimental effect of the putrefaction products ammonia, phenols, indoles, amines, sulfides, and N-nitroso compounds is now fairly acknowledged, as they are involved in systemic toxicity, nephrotoxicity, and carcinogenesis (Blachier et al., 2010; Russell et al., 2013; Barrios et al., 2015; Kobayashi, 2017).

A metataxonomic 16S rRNA gene survey of cultures of human gut microbiota in a medium where proteins and amino acids were the sole fermentable substrates recently identified many bacterial taxa that thrived, taking advantage of protein breakdown as primary or secondary degraders (Amaretti et al., 2019). Enterobacteriaceae, Sutterellaceae, and Desulfovibrionaceae, including *Escherichia–Shigella*, *Sutterella*, *Parasutterella*, and *Bilophila*, grew especially in the cultures with low inoculation load. Lachnospiraceae, Eubacteriaceae, Oscillospiraceae (formerly Ruminococcaceae), and Peptostreptococcaceae also encompassed many taxa that significantly expanded, such as *Anaerotruncus*, *Dorea*, *Oscillibacter*, *Eubacterium oxidoreducens*, *Lachnoclostridium*, *Paeniclostridium*, and *Romboutsia*.

In the current study, we performed volatilome analyses of these protein-based cultures of gut microbiota to observe the evolution of volatile organic compounds (VOCs) derived by bacterial proteolytic metabolism. Bioinformatic and chemometric analyses were performed to bridge the datasets of 16S rRNA survey, gas chromatogram (GC) MS metabolomics, and metagenome reconstruction. The aim of the current work was to complete the previous study (Amaretti et al., 2019) that identified protein degraders, providing an overall view on how gut microbiota components concur to protein fermentation. PICRUSt2 software was utilized for the inference of the functional profile communities in enrichment cultures, which was compared with that of the founding microbiota with the linear discriminant analysis-based algorithm LEfSe (Segata et al., 2011; Douglas et al., 2020). In order to evaluate the effect of subject, incubation time, culture dilution, and their interactions on the evolution of microbiota cultures, a joint dataset obtained by fusing volatilome and microbiome profiles was analyzed by means of ANOVA simultaneous component analysis (ASCA), an extension of ANOVA specifically designed for the analysis of multivariate datasets (Smilde et al., 2005; Ashrafi et al., 2020; Firmani et al., 2020; Zhou et al., 2020; Calvini et al., 2022). Such chemometric approach has been applied for the first time to interpret the results of microbiota experiments and to establish associations between specific bacterial taxa and metabolites.

## Materials and Methods

### Fermentation Experiments

Bioreactor batch fermentations were carried out in a previous study with the feces of five healthy subjects following omnivorous western diet (volunteers V1, V3, V4, V5, and V6), utilizing a protein-based medim (Amaretti et al., 2019). Briefly, the cultures were inoculated with 1 or 0.05% feces (w/v), respectively referred to as C (i.e., concentrated) or D (i.e., diluted) process, and were incubated at 37°C under a CO_2_ atmosphere, with the pH kept constant at 6.8 by automatic titration. A single preliminary fermentation run was carried out with subject V1, seeded with a C inoculation. Parallel fermentation runs were carried out with C and D inoculation conditions for the other subjects. Samples were collected at 0, 6, and 12 h of incubation and stored at −80°C until analyses.

### Chemical Analysis

The profile of VOCs was determined by solid-phase microextraction (SPME) followed by GC–MS analysis. A divinylbenzene/carboxen/polydimethylsiloxane fiber (DVB/CAR/PDMS Supelco; Sigma-Aldrich, St. Louis, MO, United States) was exposed for 1 h at 60°C to the headspace of a 10-ml vial containing 2 ml of the sample and supplemented with 10 μl of 10 M of HCl. The analyses were performed in duplicate. The volatiles were released through thermic desorption at 240°C in the injector of a GC–MS apparatus (7820–5975; Agilent Technologies, Santa Clara, CA, Untied States) equipped with a DB-624 column (30 m × 250 μm × 1.4 μm, Agilent Technologies). Separation was carried out with 1.3 ml/min of helium and applying the following thermal gradient: 2 min at 50°C, 6°C/min increase to 110°C, 10°C/min increase to 240°C, and 4 min at 240°C. The tentative assignment of the volatiles was based on the comparison of mass spectra with data of the National Institute of Standards and Technology mass spectral library (NIST 14). The peak areas of volatile compounds were taken to be their relative abundances.

### Microbiome Function Prediction

The 16S rRNA gene sequences were downloaded from National Center for Biotechnology Information (NCBI) Sequence Read Archive (SRA) repository, with BioProject ID: PRJNA540787. The sequences were processed with the QIIME2 pipeline (version 2019.1), using the plugin Vsearch for closed-reference picking (similarity threshold of 0.97) with SILVA SSU database 132 as reference for the (Amaretti et al., 2019). The feature table of the operational taxonomic units (OTUs) and their abundance across each sample were used to reconstruct the metagenome and infer the microbial functions through PICRUSt2 (version 2.3.0-b) (Douglas et al., 2020). The script picrust2_pipeline.py with default options was utilized to predict functional profiles in terms of Kyoto Encyclopedia of Genes and Genomes (KEGG) Orthology (KO) abundances. The script pathway_pipeline.py with the option ‘‘--no_regroup’’ was utilized to reconstruct metabolic modules and pathways. The differential abundance of predicted KOs characterizing C and D cultures at the different time-points was analyzed by means of linear discriminant analysis effect size (LEfSe) algorithm^1^ (Segata et al., 2011). KEGG Mapper^2^ was utilized to reconstruct the metabolic modules and pathways.

### Chemometrics

**FIGURE 1:**
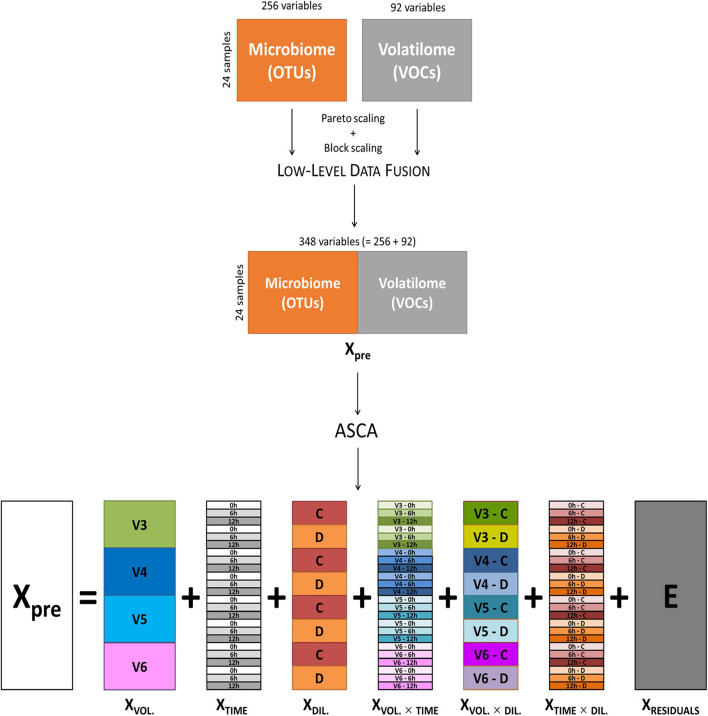
Schematic representation of low-level data fusion of microbiome and volatilome data, and ASCA decomposition of fused dataset. ASCA, ANOVA simultaneous component analysis.

Data of volatilome (i.e., the abundance of 85 VOCs) and microbiome (i.e., 221 OTUs) from cultures inoculated with V3, V4, V5, and V6 were merged by means of a low-level data fusion approach (Borràs et al., 2015; Calvini et al., 2016). Prior to data fusion, both volatilome and microbiome data were preprocessed using Pareto scaling (van den Berg et al., 2006) and block scaling (Orlandi et al., 2019). Then, low-level data fusion simply consisted in concatenating in sequence the two preprocessed data blocks into a fused dataset (*Xpre*), as sketched in the top part of Figure 1. Cultures inoculated with V1 were excluded from the analysis, since only the C cultures were available for this subject. The fused dataset was interpreted as if it resulted from a factorial experimental design with three main factors: (i) subject, with four levels (i.e., the volunteers); (ii) incubation time, with three levels (i.e., 0, 6, and 12 h); and (iii) inoculum concentration, with two levels (i.e., C and D).

ANOVA simultaneous component analysis was then used to decompose *Xpre* into effect matrices accounting for the variability induced by the volunteers (*X_VOL._*), incubation time (*X_TIME_*), and culture dilution (*X_DIL._*) as main factors; their binary interactions (*X_VOL. × TIME_*, *X_VOL. × DIL._*, *X_TIME × DIL._*); and a residuals matrix (*E*) accounting for variability not included in the model (Figure 1). The effect matrix of each factor has as many rows as the number of samples, and all the rows corresponding to samples belonging to the same factor level contain the average response of the considered level. The effect matrices of the interactions were calculated using the same procedure after a deflation step, i.e., after subtracting the effect matrices of the main factors from *Xpre*. The extent of the contribution of each factor and of each interaction to total data variance was calculated using the sum of squares of the elements of the corresponding effect matrix, and the relevant significance was assessed with a permutation test (Vis et al., 2007; Calvini et al., 2022), considering 1,000 random permutations.

For each factor and interaction, principal component analysis (PCA) was applied to the corresponding effect matrix to obtain a PCA submodel that allowed to maximize the differences between level averages, without considering the variability within samples belonging to the same level. The number of PCs considered for each PCA submodel is equal to the number of levels of the corresponding factor minus one. Scores and loadings obtained from the PCA submodels were then evaluated as in standard PCA; however, the great advantage of ASCA consists in the possibility of separately analyzing the variations ascribable to the main experimental factors and their interactions, with a gain in the interpretation of the results.

In the loading plot of a PCA submodel, the distance of a given variable from the axis origin can be used to quantify the contribution of that variable to the PCA submodel itself, to define how much that variable is important to describe the effect accounted by the PCA submodel. As an example, considering the PCA submodel of the time effect matrix, the variables with the highest distance from the origin are those showing a greater variation due to incubation time, regardless of the other factors. Based on this criterion, for each PCA submodel, only the variables with a distance from the origin higher than 0.15 (normalized loading units) were selected. Furthermore, since variables with a similar behavior are grouped together in the loading space, the selected variables were subjected to agglomerative hierarchical cluster analysis using Ward’s method (Ward, 1963) and considering Euclidean distance as metric. The resulting dendrogram allowed to highlight grouping of variables with similar behavior, i.e., correlated to each other, regardless of their nature of microbiome or volatilome data, and regardless of the number of principal components of the submodel.

Furthermore, to highlight the relationship between variables and factor levels, for each PCA submodel, a biplot jointly reporting both the score plot (related to factor levels) and loading plot (related to microbiome and volatilome variables) was used (Bro and Smilde, 2014; Bertinetto et al., 2020). In the biplot, the variables positioned in the same direction of a given level with respect to the axis origin are correlated with positive sign with the level; i.e., they show high values for that level. For example, considering the PCA submodel of incubation time effect, if indole is positively correlated with the 12 h level, it means that after 12 h of incubation, indole has increased. Similarly, a variable positioned in the opposite direction to a given level with respect to the axis origin is correlated with negative sign with the effect level (i.e., for that level, the variable has the minimum value), while if the variable direction is orthogonal to the level direction, the level has a negligible effect on the variable. Therefore, the cosine of the angle between each variable and each level can be used to quantify their correlation: values close to 1 will indicate positive correlations, values close to −1 will indicate negative correlations, and values close to 0 will be obtained for absence of correlation.

To visualize correlation information in the dendrograms of the PCA submodels, a number of circles equal to the number of levels was reported close to each variable, whose color corresponds to the cosine values: a dark red color indicates a positive correlation, a dark blue color indicates a negative correlation, and light colors/white indicate scarce/absent correlation, as codified by the corresponding color bar. Furthermore, to indicate also the importance of each variable for the considered submodel, the size of the circles for each variable is proportional to the distance of the variable from the axis origin: the larger the circles, the greater the variation of that variable due to the considered effect.

Data preprocessing and data analysis with ASCA were performed using the PLS_Toolbox software (version 8.8.1; Eigenvector Research Inc., Wenatchee, WA, United States) running under MATLAB environment (R2020b; The Mathworks Inc., Natick, MA, Untied States). The dendrograms were built using *ad hoc* routines developed in MATLAB environment.

## Results

### Volatile Organic Compounds

**FIGURE 2:**
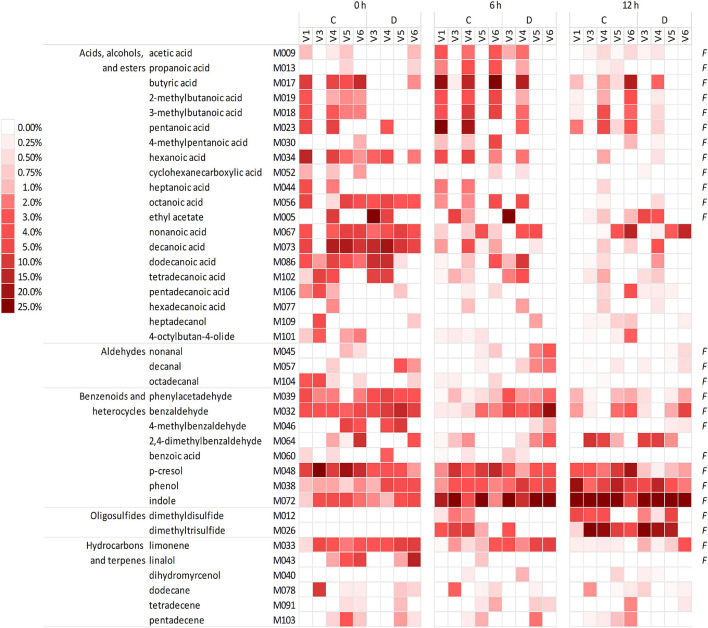
Heatmap of the main VOCs identified in the headspace of microbiota cultures at 0, 6, and 12 h. The shades of red indicate the relative abundance in the gas chromatogram. Only the VOCs occurring at least in five samples and at least once ≥1% are reported. *F* indicates the natural occurrence in the feces of healthy humans, according to de Lacy Costello et al. (2014). VOCs, volatile organic compounds.

The headspace of the microbiota cultures at 0, 6, and 12 h yielded a total of 101 VOCs, 39 of which occurred in at least five samples, with a peak contributing at least once for ≥1% of the chromatogram (Figure 2 and Supplementary Datasheet 1). The VOCs occurring most frequently and abundantly at 0 h were benzene derivatives (e.g., *p*-cresol, benzaldehyde, benzeneacetaldehyde, and phenol), indole, organic acids (butanoic, 2- and 3-methyl butanoic, pentanoic, hexanoic acids, other fatty acids with chain up to C15), and limonene. Indole and sulfides (i.e., dimethyl and trimethyl sulfides that were initially negligible) progressively increased and dominated the volatilome at 12 h. Over time, some benzene derivatives decreased in relative abundance (e.g., *p*-cresol, benzaldehyde, 4-methyl benzaldehyde, and benzeneacetaldehyde), while others increased (phenol and 2,4-methylbenzaldehyde). Terpenes, fatty acids, and SCFA tended to decrease, with some SCFA (such as acetic, propanoic, butyric, and methylbutanoic acids) that peaked at 6 h and decreased at 12 h.

### ANOVA Simultaneous Component Analysis Model

The evolution of the 16S rRNA gene profile in the set of microbiota cultures analyzed in the present study, among those described by Amaretti et al. (2019), is reported in Supplementary Figure 1. ASCA was applied to the fused dataset of volatilome and microbiome profiles to evaluate the effect of subject, incubation time, culture dilution, and their binary interactions on the evolution of protein-based microbiota cultures (Supplementary Datasheet 1). All the factors and their interactions significantly affected microbiota composition and volatilome profile (*p* < 0.05), the greatest source of variance being the subject (30.8%), followed by time (21.2%) and interaction subject × time (20.5%) (Table 1). Interindividual variations in the founding microbiota exerted the greatest effect in shaping the evolution of both the bacterial community and VOC profile. VOCs and bacterial taxa similarly correlating with subjects and with subjects across time are displayed in the dendrograms of ASCA results as reported in Supplementary Figure 2.

**TABLE 1:** Results obtained from ASCA applied on the fused dataset of microbiome and volatilome data.

**Factor**	**Effect (%)**	***p*-Value**
Subject	30.8	0.001
Time	21.2	0.001
Dilution	6.6	0.001
Subject × time	20.5	0.001
Subject × dilution	8.5	0.001
Time × dilution	4.2	0.007
Residual	8.2	

*ASCA, ANOVA simultaneous component analysis.*

**FIGURE 3:**
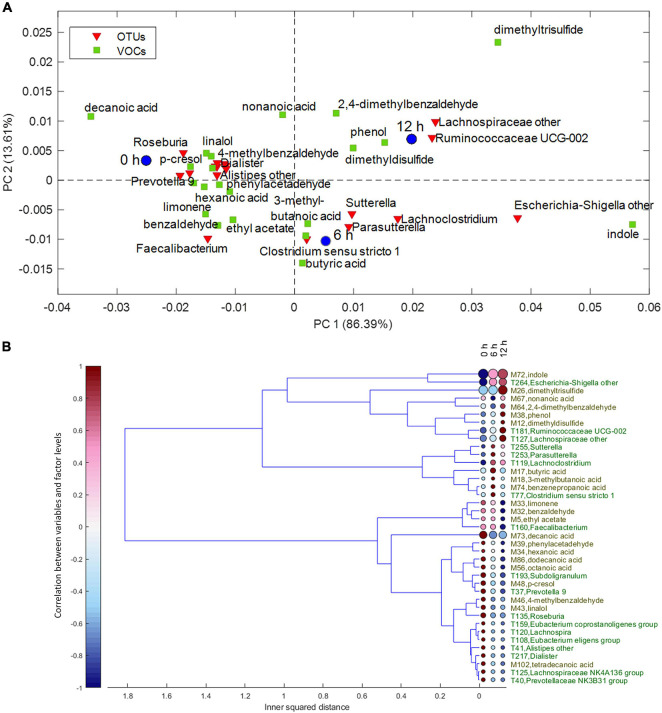
**(A)** Biplot of the PCA submodel of time effect matrix, where only the selected variables are displayed. **(B)** Dendrogram of time effect matrix, where for each variable the size of the circles reflects its importance for the submodel, while colors of the circles represent the correlations between the variable and each level of time factor. PCA, principal component analysis.

Despite the interindividual differences, relevant associations of VOCs and bacteria increasing or decreasing during the enrichment in the protein-based medium were observed. For the factor time, the result of ASCA is displayed in the PCA biplot of Figure 3A. The corresponding dendrogram of variables, computed as described in *Chemometrics*, is reported in Figure 3B. Butyric, 3-methyl butanoic, and benzenepropanoic acids among the VOCs and some Lachnoclostridiaceae (*Lachnoclostridium*), Clostridiaceae (*Clostridium sensu stricto*), and Sutterellaceae (*Sutterella* and *Parasutterella*) among the bacterial taxa were major determinants of cultures at 6 h and remained stable or slightly decreased at 12 h. Increasing levels of indole and *Escherichia–Shigella* characterized cultures at 6 and 12 h; while 2,4-dimethyl benzaldehyde, phenol, dimethyl disulfide, Ruminococcaceae UCG-002, and unclassified Lachnospiraceae were major determinants only at 12 h.

On the other hand, some VOCs and bacterial taxa characterized samples at 0 h and decreased in the subsequent samples, while others remained stable for 6 h and decreased only at 12 h. Fatty acids and benzene derivatives (such as benzaldehyde, phenylacetaldehyde, 4-methyl benzaldehyde, and *p*-cresol) decreased at 6 or 12 h, with some Lachnospiraceae [*Roseburia*, *Lachnospira* (*Eubacterium*) *eligens*], Oscillospiraceae (i.e., Ruminococcaceae) (*Subdoligranulum* and *Faecalibacterium*), Eubacteriaceae (*Eubacterium coprostanoligenes*), Veillonellaceae (*Dialister*), Rikenellaceae (*Alistipes*), and Prevotellaceae (*Prevotella*).

The analysis of the interaction between subject and incubation time (Supplementary Figure 2D) revealed that indole, dimethyl trisulfide, and *Escherichia–Shigella* were strongly associated in a cluster separate from the other variables. These variables presented a similar increasing trend in all the subjects, particularly in subjects V3 and V5. Another cluster encompassed variables that presented an increasing trend in the V3–V5 subjects, particularly in V4: the taxa *Dorea*, *Ruminiclostridium*, Lachnospiraceae UCG-004, *Akkermansia*, *Allisonella*, and unclassified Ruminococcaceae and Lachnospiraceae were associated with the dimethyl disulfide, phenol, benzenepropanoic acid, and 3-methylbutanoic acid.

The taxa *Lachnoclostridium*, *Acidaminococcus intestini*, and Ruminococcaceae UCG-002 shared a common increasing trend, particularly in subjects V5 and V6, but were not associated with any VOCs. Likewise, *Parasutterella* and *Bacteroides*, which increased only in subject V6, and *Sutterella*, which slightly increased in V3 and V4, were not associated with any VOCs. All the other clusters encompassed variables that shared a similar tendency to decrease, more or less accentuated according to the subject. For example, a vast cluster of variables shared a general tendency to decrease, less evident in V3. Within this cluster, *Faecalibacterium*, *Subdoligranulum*, and *Barnesiella* were associated with butyric and octanoic acids.

The analysis of the inoculum concentration (Supplementary Figure 2C) and of its interaction with time (Supplementary Figure 2F) pointed out relationships characterizing concentrated or diluted cultures. In particular, Supplementary Figure 2F shows that *Escherichia–Shigella* and indole showed a most significant increase with time in D cultures, while a variety of taxa and VOCs increased more throughout cultivation in C cultures, such as unclassified Ruminococcaceae and Lachnospiraceae, *Akkermansia*, *Lachnoclostridium*, dimethyl trisulfide, dimethyl disulfide, phenol, butyric, methylbutanoic, and pentanoic acids. A cluster of variables, such as *Bacteroides*, *Clostridium sensu stricto*, and benzenepropanoic acid, decreased during cultivation in C cultures, but had a positive correlation with grown D cultures. The decrease of *Alistipes* and *p*-cresol was less marked in C cultures than in the corresponding D ones. The remaining variables that show a decrement throughout cultivation behave alike in C and D cultures and lay in the same vast cluster.

### Predicted Metabolic Functions

**FIGURE 4:**
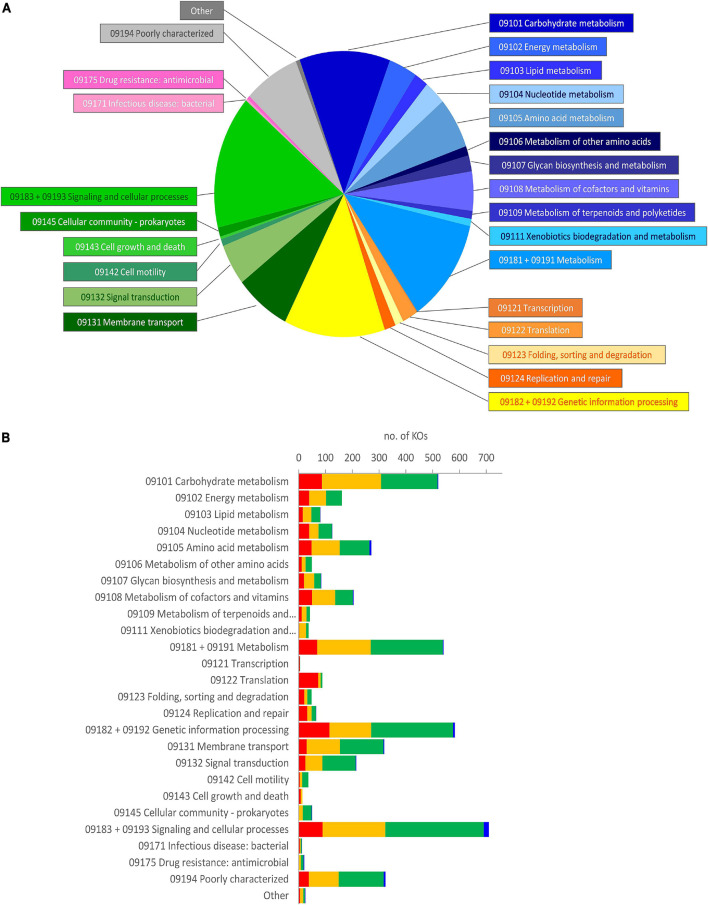
Brite’s hierarchical classification of the functions predicted in the metagenome of microbiota cultures, enriched in a protein-based medium. **(A)** Classification of the core KOs shared by all the founding microbiota at 0 h. Blue, metabolism; yellow/orange, genetic information processing; green, environmental information processing and cellular processes; pink, human diseases; gray, poorly characterized and other functions. **(B)** Distribution of the KOs that presented a significant differential abundance in enrichment cultures, according to LEfSe (*p* < 0.05). The KOs in the core metagenome that decreased (red), did not significantly change (yellow), or increased (green), and those, absent in the core metagenome that appeared during cultivation (blue), are reported. KO, Kyoto Encyclopedia of Genes and Genomes Orthology.

The metagenome of the microbiota cultures was reconstructed based on the metataxonomic 16S rRNA gene profile. The functions therein encoded were predicted with PICRUSt2, which yielded a total of 6,301 functional orthologs assigned in KO database (Supplementary Datasheet 2). The five founding microbiota, utilized to inoculate the cultures, harbored 5,894 KOs (5,015–5,302 per sample), with the vast majority of them being encompassed in an initial core of 4,566. According to Brite’s hierarchical classification of the 4,566 common functions, the core KOs were distributed among metabolism (2,098), genetic information processing (781), and signaling and cellular processes (1,318) (Figure 4A). The core KOs with a predicted metabolic function were attributed to 226 metabolic modules, among which 122 were complete, 29 lacked one block (i.e., reaction), and 22 lacked two blocks (Supplementary Datasheet 3). Five hundred thirty-eight out of the 2,098 metabolic genes encoded for functions that were not encompassed in the modules or pathways of KEGG, including 97 proteases/peptidases. Among the 1,318 KOs involved in signaling and cellular processes, 627 were classified within a vast array of gene sets for PTS and ABC transporters (Supplementary Datasheet 2), while the remaining 691 encoded for several other transporters, and signaling and cellular functions not included in KEGG maps.

During the cultivation, the number of predicted KOs did not significantly change (*p* > 0.05), despite that the richness and the evenness of the microbial community decreased, more evidently in D cultures (Amaretti et al., 2019; Supplementary Figure 3). LEfSe analysis pointed out 2,180 KOs as biomarkers of protein-enriched cultures (Supplementary Datasheet 2). These KOs presented a significant differential abundance at 6 or 12 h compared with 0 h in C or D cultures. Most of the KOs identified as biomarkers (2,114) were present among the core KOs, while only 66 were absent and appeared in grown cultures. On the other hand, 873 KOs (837 belonging to the initial core) were biomarkers of cultures at 0 h and then significantly decreased. The biomarker KOs were spread in all the main Brite’s functional groups that were observed at 0 h; hence, the relative weight of functional groups was not greatly affected (Figure 4B).

**FIGURE 5:**
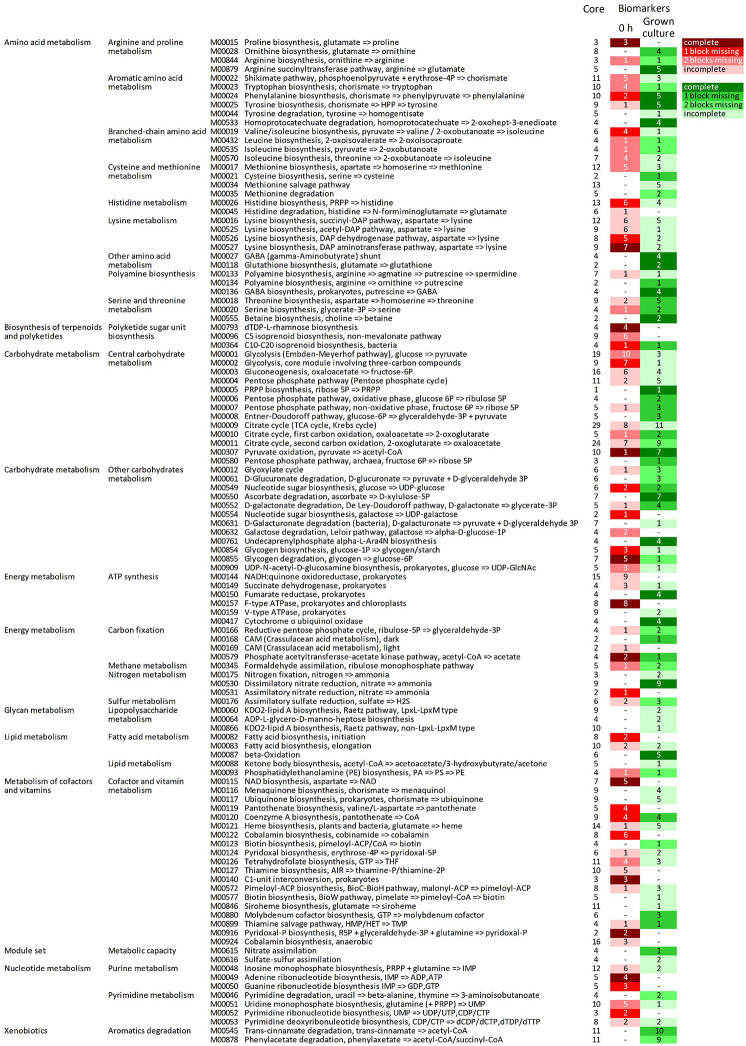
Complete metabolic modules predicted in the core metagenome at 0 h presenting statistically significant differential abundance between 0 h and grown cultures (6 or 12 h). For each module, the total number of KOs recognized by KEGG’s mapper and the number of biomarker KOs characterizing cultures at 0 h (red shades) and grown cultures (green shades), according to LEfSe, are reported. Red and green shades from the darkest to the lightest indicate the degree of completeness of the module changing in abundance (complete, 1 block missing, 2 blocks missing, and >2 blocks missing); “–” indicates non-significant changes. KEGG, Kyoto Encyclopedia of Genes and Genomes; KO, KEGG Orthology.

The predicted KOs that increased in relative abundance during growth were involved in many modules (164 out of 226), 92 of which were initially complete (Figure 5, Supplementary Datasheet 3 and Supplementary Figure 4). Fifty-two complete metabolic modules presented an increase of all, all but one, or all but two blocks (16, 20, and 16 modules, respectively). These were implicated in central carbohydrate and energy metabolism (15 and 8, respectively), degradative or anabolic pathways of amino acids (18), lipid and terpenoid metabolism (three), cofactors and vitamins metabolism (three), nucleotide metabolism (one), aromatics degradation (two), and nitrate assimilation (one).

Among the predicted modules that expanded, those involved in central carbohydrate and energy metabolism included Entner–Doudoroff glycolysis, the pentose phosphate shunt, the glyoxylate cycle, and portions of the tricarboxylic acid (TCA) cycle. The modules of amino acid degradation that were predicted to increase were involved in methionine breakdown and in interconversions between glutamate, ornithine, arginine, and putrescine, and their channeling to GABA, and finally toward succinyl-CoA. Degradative modules of aromatic amino acids yielding succinyl-CoA or acetyl-CoA *via* homoprotocatechuate, trans-cinnamate, or phenylacetate degradation pathways were also reconstructed. On the other hand, modules of the biosynthetic pathways (e.g., those yielding serine and cysteine, branched-chain amino acids, chorismate and aromatic amino acids, and threonine) were also predicted to increase. Among predicted transport systems, complete PTS systems for various sugars and ABC transporters for nitrite/nitrate, urea, oligopeptides, and some amino acids (e.g., glycine/proline, aspartate/glutamate/glutamine, spermidine/putrescine, and cystine) significantly increased (Supplementary Figure 5).

The predicted KOs that decreased in relative abundance affected 124 metabolic modules, 71 of which were initially complete (Figure 5). Forty-four complete reconstructed modules were characterized by a decrease in all, all but one, or all but two the blocks (11, 16, and 17 modules, respectively). In the central carbohydrate and energy metabolism, many blocks within the Embden–Meyerhof pathway and the core module of glycolysis involving three-carbon compounds, parts of TCA cycle, and F-type ATPase were predicted to significantly decrease. Many anabolic modules were also predicted to be negatively affected, such as those yielding fatty acids, cofactors and vitamins, purines and pyrimidines, amino and nucleotide sugars, and various amino acids (e.g., proline, lysine, histidine, aromatic, and branched-chain amino acids).

## Discussion

Protein putrefaction by gut bacteria is known to release an array of harmful metabolites. In the present study, the volatile metabolites released by cultures of human gut microbiota, where peptides and proteins were supplied as a sole fermentable substrate, were determined and analyzed with a chemometric approach, to establish a relationship with the microbial taxa that got enriched during cultivation. The cultures herein described yielded ammonia, acetate, propionate, and butyrate throughout the first 12 h of incubation, at a higher or lower rate depending on the concentration of the inoculum (Amaretti et al., 2019).

The headspace of cultures at 0 h were a mixture of linear and branched SCFA, aromatic compounds, aldehydes, and terpenes that normally compose to the fecal volatilome of the stools of healthy subjects, of both microbial and dietary origins (Garner et al., 2007; de Lacy Costello et al., 2014). Fatty acids and derivatives with chain longer than octanoic acid derived from dehydrated bile were utilized in the medium (data not shown). The cultivation on a protein-based medium of the gut microbiota determined a change in the VOC profile, characterized by the progressive decrease in relative abundance of the organic acids longer than pentanoic acid, some benzenoids initially abundant (such as cresol, benzaldehyde, and phenylacetaldehyde), aldehydes, and hydrocarbons. These compounds tended to disappear during the fermentation, being degraded and channeled into fermentative pathways or overwhelmed by other metabolites that progressively became dominant. Indeed, VOC quantification is representative of the relative abundance in culture headspace due to the intrinsic properties of SPME–GC technique, which is affected by saturation of the absorption sites of the fiber and competitive displacement of the analytes (Dixon et al., 2011).

On the other hand, the headspace of cultures got enriched with linear and branched fatty acids with chain of two to five carbons mainly in the first 6 h, and with oligosulfides (dimethyl disulfide and dimethyl trisulfide), indole, and phenol throughout the 12 h of incubation. The organic acids eventually originate from an array of TCA cycle intermediates, pyruvate, and phosphate- or coenzyme A-linked SCFA derivatives, which are produced from bacterial deamination or dissimilatory transformations (such as Stickland fermentation) of amino acids (Oliphant and Allen-Vercoe, 2019). Acetic, propionic, and butyric acids are the main end products of many amino acids fermentations; while branched-chain fatty acids (BCFAs), such as 2-methylbutanoic, 3-methylbutanoic, and 4-methylpentanoic acids, are principally produced by gut microbial action on branched amino acids (Bäckhed et al., 2005; Fan et al., 2015).

At 12 h, the organic acids were overwhelmed by oligosulfides, indole, and phenol generated in increasing amount throughout the fermentation and progressively becoming dominant. The oligosulfides increased in all the samples, generated from sulfur-containing amino acids. These metabolites are commonly detected in feces and putrefied matrices, including carrions and dung, but the metabolic route yielding them in the colonic ecosystem has not been clarified so far, unlike in other environments (Statheropoulos et al., 2007; Liu et al., 2013; de Lacy Costello et al., 2014). Cysteine and methionine fermentation by the gut microbiota yield hydrogen sulfide and methanethiol, which, in turn, combine into sulfides (Smith and Macfarlane, 1997; Tangerman, 2009). Phenol and indole accumulated throughout the process as a result of tyrosine and tryptophan catabolism, respectively (Smith and Macfarlane, 1997). A wide diversity of benzene and phenol derivatives can derive from the degradation of phenylalanine and tyrosine, through metabolic routes that are initiated by deamination or decarboxylation and that share some phenylpropanoids (e.g., cinnamate and coumarate) with the transformation of dietary phytochemicals (Russell et al., 2013; Oliphant and Allen-Vercoe, 2019). Indole is the major bacterial metabolite of tryptophan that undergoes more rarely to decarboxylation or deamination by the intestinal bacterial community (Williams et al., 2014; Oliphant and Allen-Vercoe, 2019).

The application of an ASCA model to a fused dataset including both volatilome and microbiome profiles highlighted strong associations between certain metabolites and specific bacterial taxa, which took advantage of the protein-based growth medium, net of the interindividual differences in the founding microbiota that were a main cause of variance affecting the evolution of both the bacterial community and VOC profile. A consistent association between indole and *Escherichia–Shigella* was identified in all the ASCA submodels, except for subject and subject × dilution effects. Some Enterobacteriaceae, including *Escherichia–Shigella*, can transform tryptophan into indole, due to the production of tryptophanase (Gao et al., 2018; Raimondi et al., 2019; Amaretti et al., 2020). Unexpectedly, such association between *Escherichia–Shigella* and indole was not found by Amaretti et al. (2019), where bacteria and indole concentrations were compared using Spearman’s rank correlation analysis, presumably due to the prevailing effect of the interindividual differences, and since indole reached rapidly high concentrations in C cultures, whereas several taxa of Proteobacteria, including *Escherichia coli*, bloomed mainly in D cultures. Such correlation was pinpointed by ASCA, which successfully decomposed the sources of variance in different factors, thus isolating the deceiving factors such as, in this case, the inoculum concentration.

ANOVA simultaneous component analysis also pointed out the association of butyric, 3-methyl butanoic, and benzenepropanoic acids with some bacterial taxa that were major determinants of cultures at 6 h, such as Lachnoclostridiaceae (*Lachnoclostridium*), Clostridiaceae (*Clostridium sensu stricto*), and Sutterellaceae (*Sutterella* and *Parasutterella*). Even though any causal relationship cannot be inferred by ASCA, it seems reasonable that these bacterial groups took part to the fermentation of linear, branched, and aromatic amino acids, at least in the early stages of the fermentation. Similarly, other unclassified Lachnospiraceae and Ruminococcaceae UCG-002 resulted in the association with the formation of oligosulfides, phenol, and 2,4-dimethyl benzaldehyde that characterized cultures at 12 h. These taxa might have been involved in the fermentation of cysteine and methionine, and/or in the rearrangement of the sulfides deriving from sulfur-containing amino acids, and in the fermentation of aromatic amino acids as well.

ANOVA simultaneous component analysis submodels taking into account the interaction of incubation time with the subjects and the concentration of the inoculum highlighted a plethora of associations that characterized specific cultures, which may be a clue of the role played by selected microbial taxa in the generation of specific metabolites, which deserves deeper investigation. For instance, members of Lachnospiraceae and Ruminococcaceae (including the genera *Dorea* and *Ruminiclostridium*, respectively), the Verrucomicrobiaceae *Akkermansia*, and the Veillonellaceae *Allisonella* were associated with the oligosulfides, phenol, benzenepropanoic acid, and 3-methylbutanoic acid.

The metagenome reconstruction and its functional annotation indicated that the founding microbiota shared the vast majority of the predicted functions, 4,566 KOs out of 5,015–5,302 KOs per sample. This great proportion of shared functions contrasts with the limited proportion of shared OTUs (70 out 324) in common across the founding microbiota at 0 h. The wide repertoire of genes common to bacterial communities with different taxonomic composition endorse the fact that gut microbiota is an ecological system based on several differentiated microorganisms that perform a similar functional role, with highly conserved gene composition and functional capacity (Moya and Ferrer, 2016; Heintz-Buschart and Wilmes, 2018; Tian et al., 2020).

The selective pressure due to sole amino acids as carbon source modified microbial composition, without affecting the richness and diversity of functional profiles. Over the 12-h growth, the number of predicted KOs did not change significantly, in the face of a decrease of richness and evenness of the microbial community (Amaretti et al., 2019), according to the main stability and resilience of the human microbiome in response to perturbations (Moya and Ferrer, 2016; Heintz-Buschart and Wilmes, 2018).

The relative abundance of several KOs significantly changed, likely affecting the sets of transcribed and expressed genes, proteins, and metabolites. As expected, some predicted metabolic modules encompassing enriched KOs had a role in amino acid degradation. Predictions showed a significant increase in the abundance of modules for amino acid catabolism, particularly those belonging to the superpathways of ornithine, arginine, and putrescine transformation to GABA and eventually to succinyl-CoA, of methionine degradation, and various routes of breakdown of aromatic compounds. The expansion of these degradative pathways was accompanied by an increase in the modules for amino acid biosynthesis or interconversions and in the transporters for amino acids and peptides, confirming that protein breakdown by “true” proteolytic makes amino acids available for assimilation by the various members of the bacterial community, which have their own capacity to incorporate the amino acids into anabolic processes and/or to ferment them to produce energy.

## Conclusion

Different multivariate analysis approaches were exploited to pinpoint the relationships among protein fermentation, metabolites, and bacteria. The fused set of microbiome and volatilome data was processed by ASCA, allowing to estimate the effects of the different factors encoded in the experimental design. The metagenome reconstruction and LEfSe comparison of encoded functions allowed the identification of predicted metabolic modules and pathways characterizing the protein degraders within the microbiota. This articulated strategy enabled the identification of unedited associations between bacteria and metabolites, shedding light on the still vast complexity of intestinal protein degradation.

## Data Availability Statement

The original contributions presented in the study are included in the article/Supplementary Material, further inquiries can be directed to the corresponding author/s.

## Ethics Statement

The studies involving human participants were reviewed and approved by the Comitato Etico Provinciale, Azienda Policlinico di Modena, Italy. The patients/participants provided their written informed consent to participate in this study.

## Author Contributions

AA, MR, and AU conceived the study. SR and AL carried out the chemical analysis. FC and AA carried out the function prediction and pathway reconstruction. RC and AU conceived and performed the ASCA model. AA wrote the manuscript with contributions from all other authors. All authors contributed to the article and approved the submitted version.

## Conflict of Interest

The authors declare that the research was conducted in the absence of any commercial or financial relationships that could be construed as a potential conflict of interest.

## Publisher’s Note

All claims expressed in this article are solely those of the authors and do not necessarily represent those of their affiliated organizations, or those of the publisher, the editors and the reviewers. Any product that may be evaluated in this article, or claim that may be made by its manufacturer, is not guaranteed or endorsed by the publisher.

## Footnotes

1
https://huttenhower.sph.harvard.edu/galaxy

2
https://www.genome.jp/kegg/mapper/reconstruct.html
