# Supplementary material for: Multivariate Analysis in Microbiome Description: Correlation of Human Gut Protein Degraders, Metabolites, and Predicted Metabolic Functions
Source: Front Microbiol. 2021 Sep 17;12:723479. doi: 10.3389/fmicb.2021.723479 (PMC8484906; doi:10.3389/fmicb.2021.723479)
Supplement: Supplementary file 4 [file Data_Sheet_1.PDF]

frontiers

Multivariate analysis in microbiome description: correlation of human gut protein degraders, metabolites and metabolic functions

Stefano Raimondi [1,#], Rosalba Calvini [1,#], Francesco Candeliere [1], Alan Leonardi [2] , Alessandro Ulrici [1,2], Maddalena Rossi [1,2], Alberto Amaretti [1,2,*]

[1] Department of Life Sciences, University of Modena and Reggio Emilia, Modena, Italy

[2] BIOGEST-SITEIA, University of Modena and Reggio Emilia, Modena, Italy

*** Correspondence:**
Alberto Amaretti
alberto.amaretti@unimore.it

Supplementary Material

Suppl. Fig. 1. Stacked bar-plot representation of microbiota compositions during C and D batch cultivation, with taxonomic features collapsed at the level of genus. The taxa that remained unclassified or never occurred with abundance higher than 2.5% are grouped as others (*). The figure is adapted from Amaretti et al. 2019.

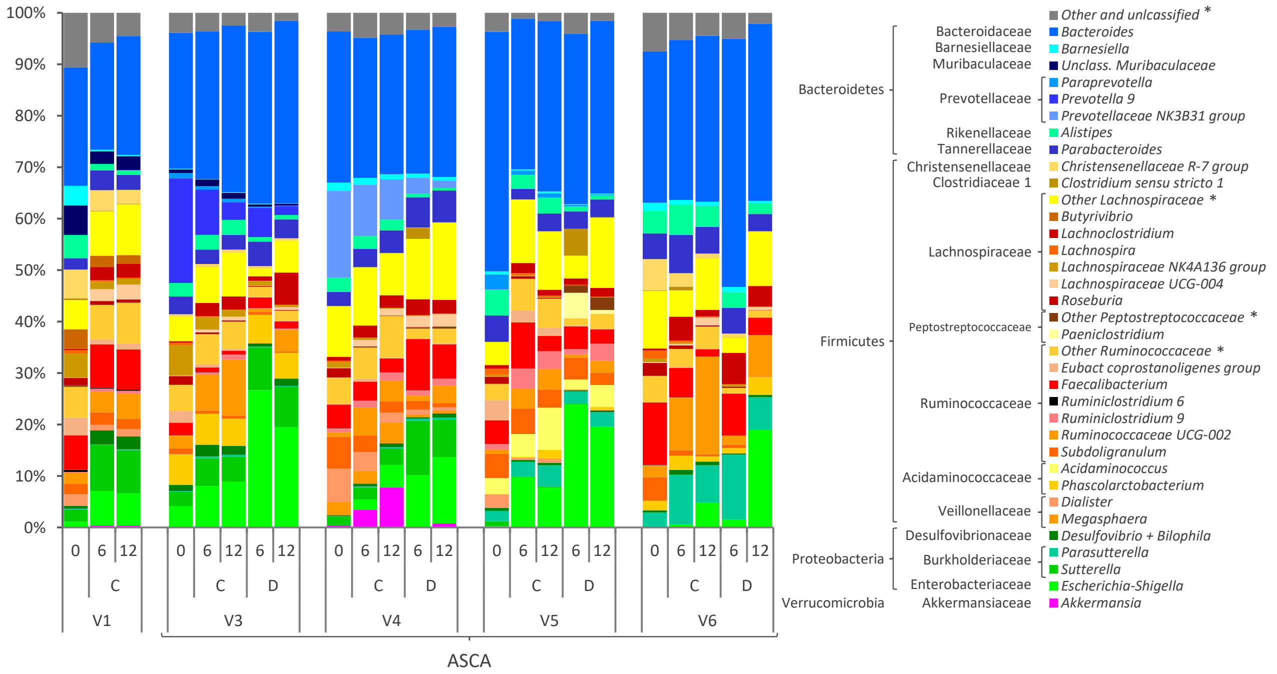

Suppl. Fig. 2. Dendrograms of ASCA submodels for the following factors and interactions: subject (A), time (B), dilution (C), subject × time (D), subject × dilution (E), and dilution × time (F).

A

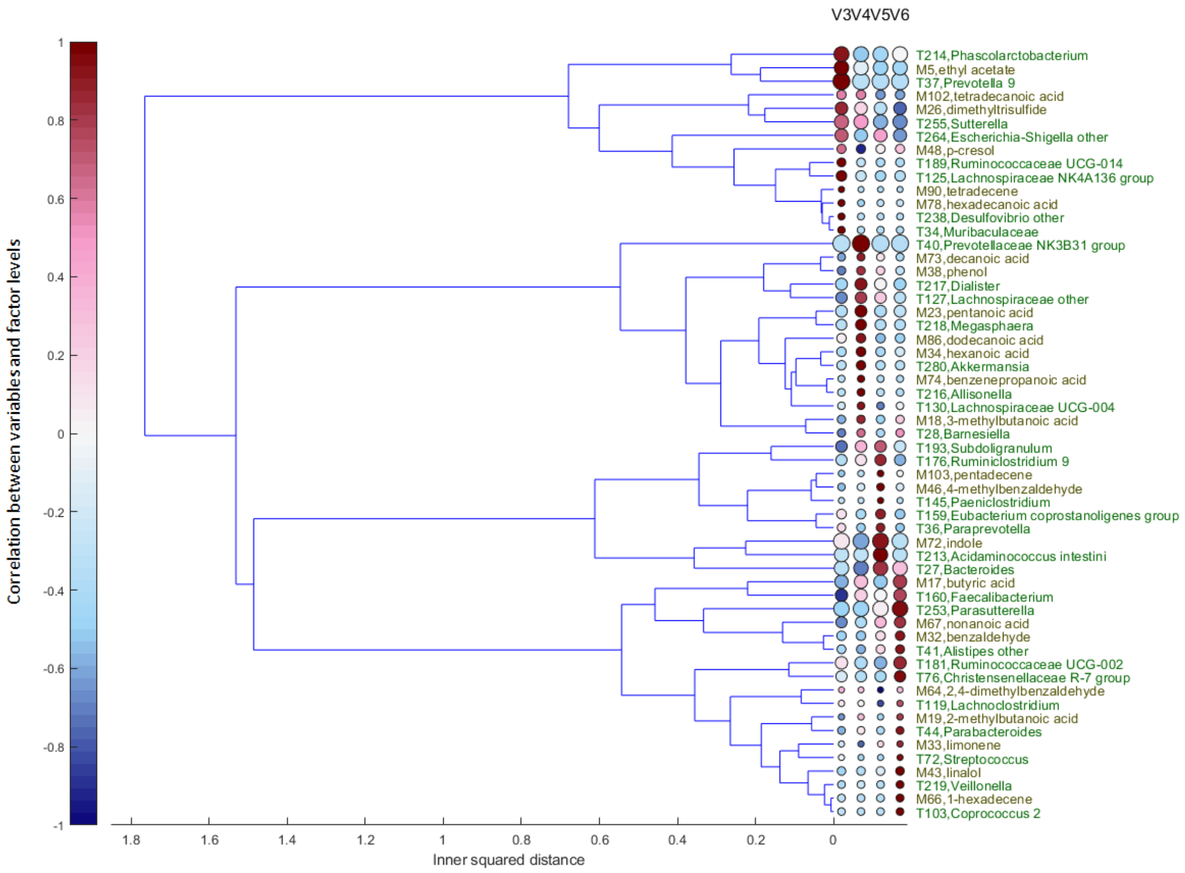

B

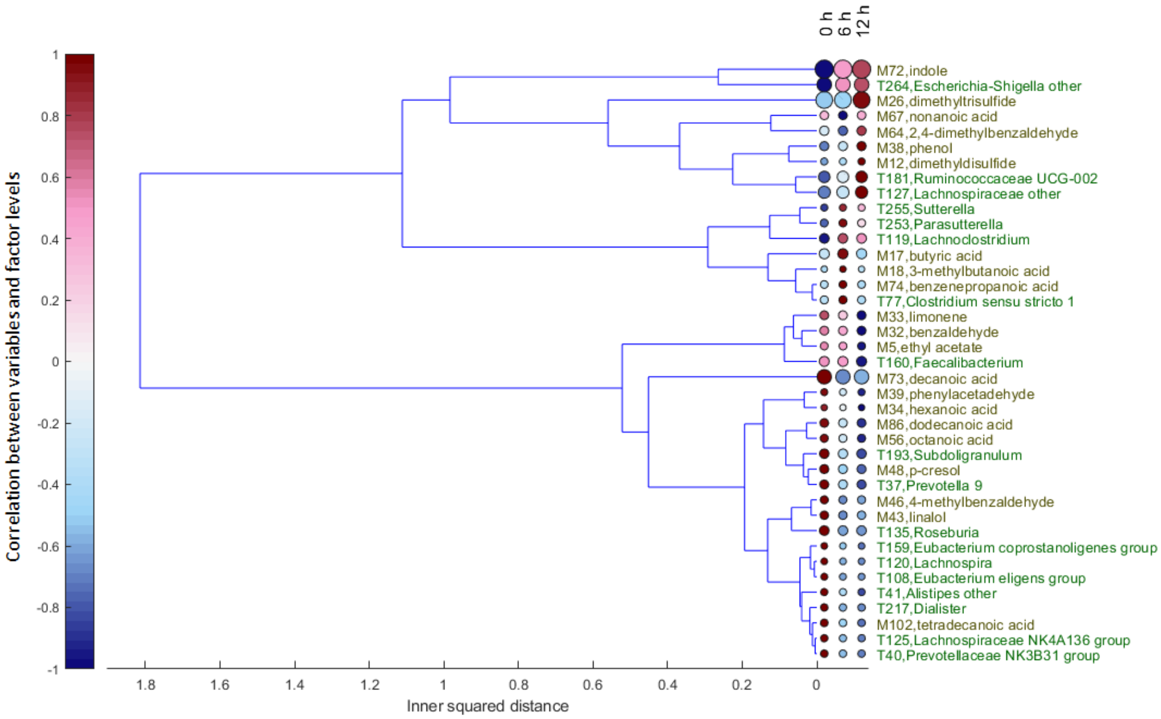

C

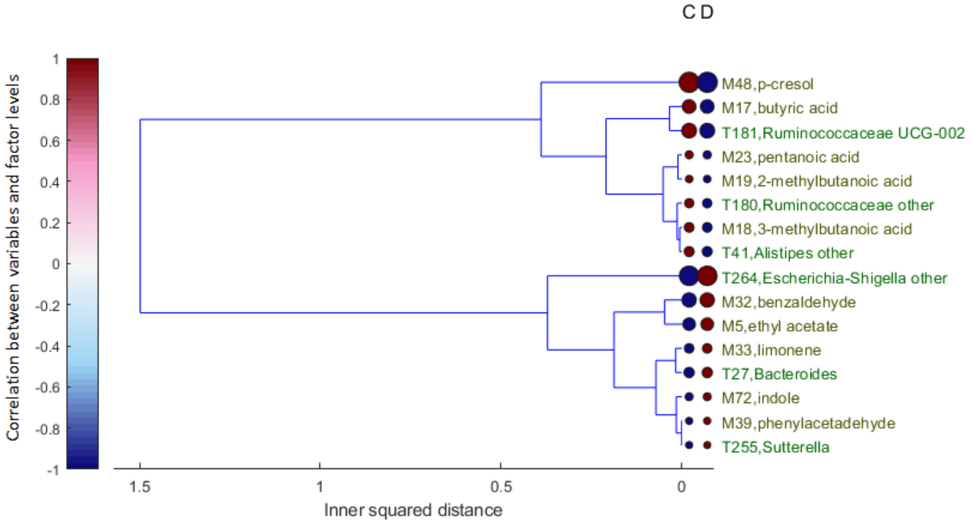
C D
Correlation between variables and factor levels
1
0.8
0.6
0.4
0.2
0
-0.2
-0.4
-0.6
-0.8
-1
M48,p-cresol
M17,butyric acid
T181,Ruminococcaceae UCG-002
M23,pentanoic acid
M19,2-methylbutanoic acid
T180,Ruminococcaceae other
M18,3-methylbutanoic acid
T41,Alistipes other
T264,Escherichia-Shigella other
M32,benzaldehyde
M5,ethyl acetate
M33,limonene
T27,Bacteroides
M72,indole
M39,phenylacetadehyde
T255,Sutterella
1.5
1
0.5
0
Inner squared distance

D

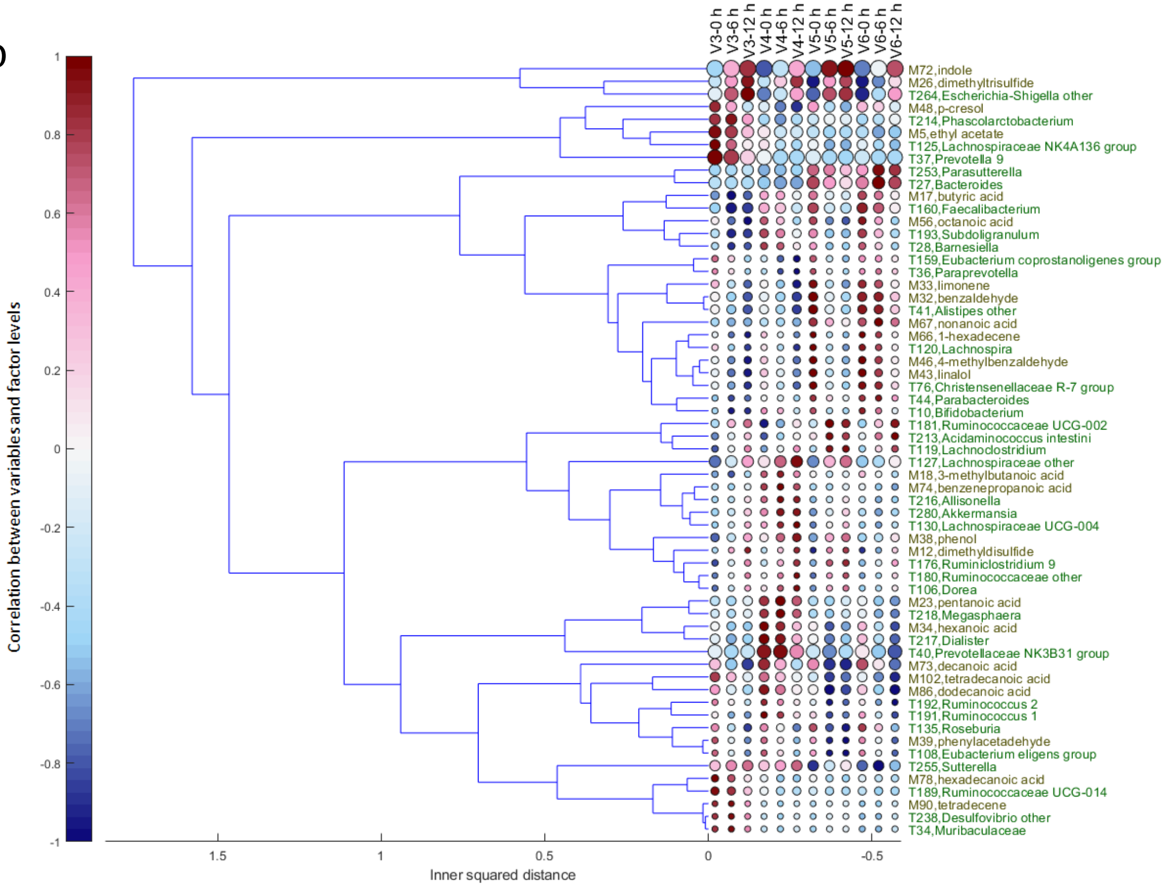
V3-0 h
V3-6 h
V3-12 h
V4-0 h
V4-6 h
V4-12 h
V5-0 h
V5-6 h
V5-12 h
V6-0 h
V6-6 h
V6-12 h
Correlation between variables and factor levels
1
0.8
0.6
0.4
0.2
0
-0.2
-0.4
-0.6
-0.8
-1
M72,indole
M26,dimethyltrisulfide
T264,Escherichia-Shigella other
M48,p-cresol
T214,Phascolarctobacterium
M5,ethyl acetate
T125,Lachnospiraceae NK4A136 group
T37,Prevotella 9
T253,Parasutterella
T27,Bacteroides
M17,butyric acid
T160,Faecalibacterium
M56,octanoic acid
T193,Subdoligranulum
T28,Barnesiella
T159,Eubacterium coprostanoligenes group
T36,Paraprevotella
M33,limonene
M32,benzaldehyde
T41,Alistipes other
M67,nonanoic acid
M66,1-hexadecene
T120,Lachnospira
M46,4-methylbenzaldehyde
M43,linalol
T76,Christensenellaceae R-7 group
T44,Parabacteroides
T10,Bifidobacterium
T181,Ruminococcaceae UCG-002
T213,Acidaminococcus intestini
T119,Lachnoclostridium
T127,Lachnospiraceae other
M18,3-methylbutanoic acid
M74,benzenepropanoic acid
T216,Allisonella
T280,Akkermansia
T130,Lachnospiraceae UCG-004
M38,phenol
M12,dimethyldisulfide
T176,Ruminiclostridium 9
T180,Ruminococcaceae other
T106,Dorea
M23,pentanoic acid
T218,Megasphaera
M34,hexanoic acid
T217,Dialister
T40,Prevotellaceae NK3B31 group
M73,decanoic acid
M102,tetradecanoic acid
M86,dodecanoic acid
T192,Ruminococcus 2
T191,Ruminococcus 1
T135,Roseburia
M39,phenylacetadehyde
T108,Eubacterium eligens group
T255,Sutterella
M78,hexadecanoic acid
T189,Ruminococcaceae UCG-014
M90,tetradecene
T238,Desulfovibrio other
T34,Muribaculaceae
1.5
1
0.5
0
-0.5
Inner squared distance

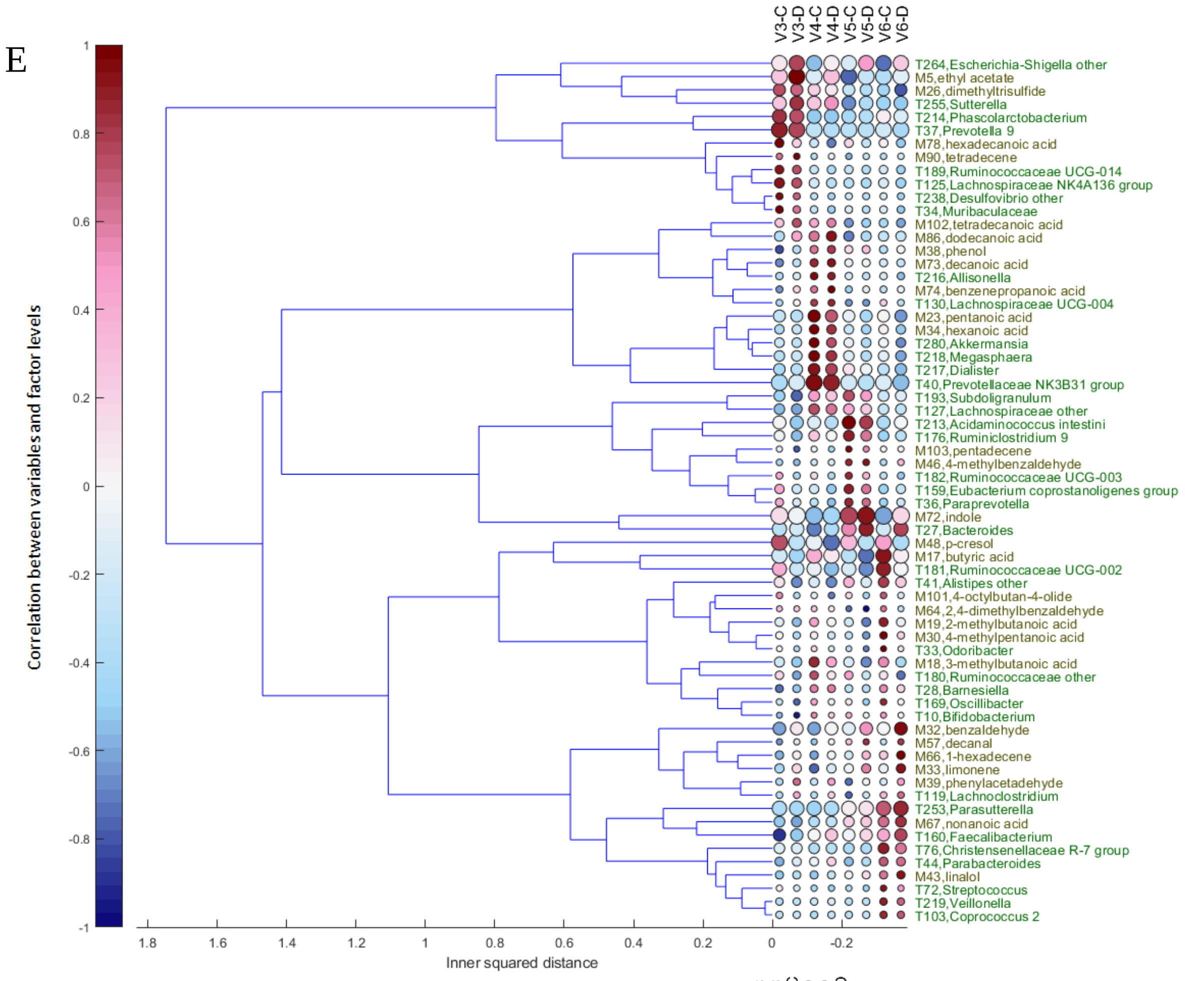
E
Correlation between variables and factor levels
1
0.8
0.6
0.4
0.2
0
-0.2
-0.4
-0.6
-0.8
-1
V3-C
V3-D
V4-C
V4-D
V5-C
V5-D
V6-C
V6-D
T264,Escherichia-Shigella other
M5,ethyl acetate
M26,dimethyltrisulfide
T255,Sutterella
T214,Phascolarctobacterium
T37,Prevotella 9
M78,hexadecanoic acid
M90,tetradecene
T189,Ruminococcaceae UCG-014
T125,Lachnospiraceae NK4A136 group
T238,Desulfovibrio other
T34,Muribaculaceae
M102,tetradecanoic acid
M86,dodecanoic acid
M38,phenol
M73,decanoic acid
T216,Allisonella
M74,benzenepropanoic acid
T130,Lachnospiraceae UCG-004
M23,pentanoic acid
M34,hexanoic acid
T280,Akkermansia
T218,Megasphaera
T217,Dialister
T40,Prevotellaceae NK3B31 group
T193,Subdoligranulum
T127,Lachnospiraceae other
T213,Acidaminococcus intestini
T176,Ruminiclostridium 9
M103,pentadecene
M46,4-methylbenzaldehyde
T182,Ruminococcaceae UCG-003
T159,Eubacterium coprostanoligenes group
T36,Paraprevotella
M72,indole
T27,Bacteroides
M48,p-cresol
M17,butyric acid
T181,Ruminococcaceae UCG-002
T41,Alistipes other
M101,4-octylbutan-4-olide
M64,2,4-dimethylbenzaldehyde
M19,2-methylbutanoic acid
M30,4-methylpentanoic acid
T33,Odoribacter
M18,3-methylbutanoic acid
T180,Ruminococcaceae other
T28,Barnesiella
T169,Oscillibacter
T10,Bifidobacterium
M32,benzaldehyde
M57,decanal
M66,1-hexadecene
M33,limonene
M39,phenylacetadehyde
T119,Lachnoclostridium
T253,Parasutterella
M67,nonanoic acid
T160,Faecalibacterium
T76,Christensenellaceae R-7 group
T44,Parabacteroides
M43,linalol
T72,Streptococcus
T219,Veillonella
T103,Coprococcus 2
1.8
1.6
1.4
1.2
1
0.8
0.6
0.4
0.2
0
-0.2
Inner squared distance

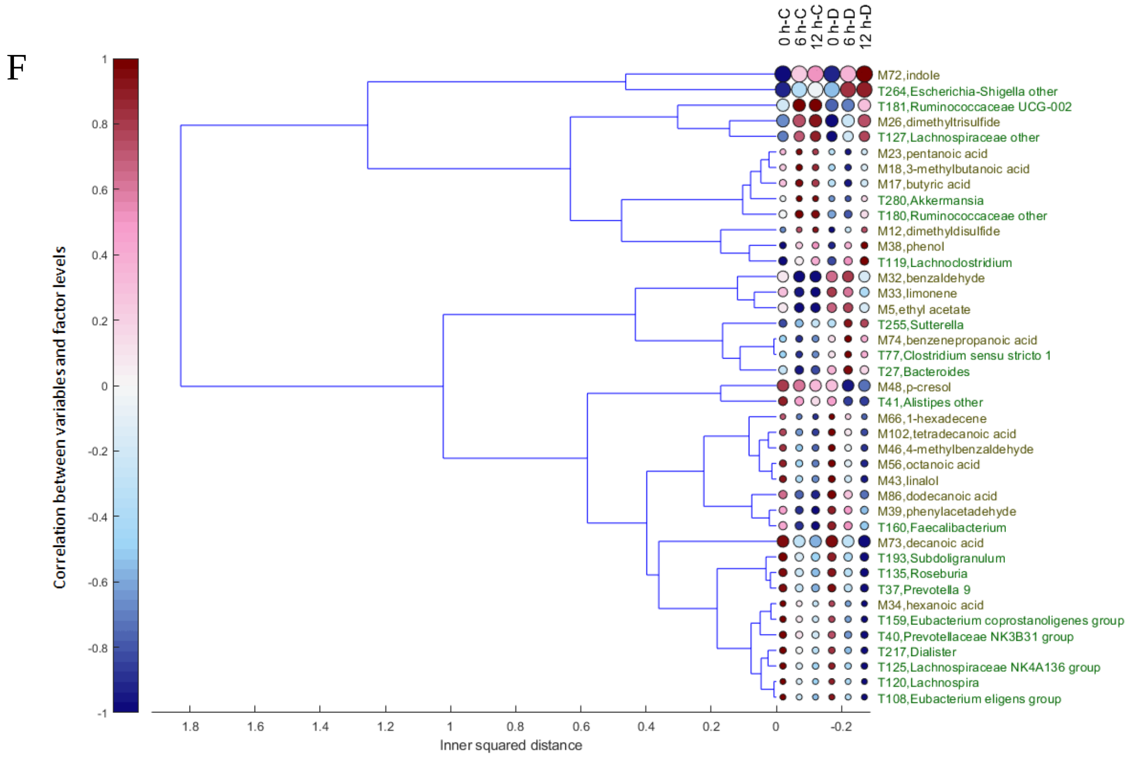
F
Correlation between variables and factor levels
1
0.8
0.6
0.4
0.2
0
-0.2
-0.4
-0.6
-0.8
-1
0 h-C
6 h-C
12 h-C
0 h-D
6 h-D
12 h-D
M72,indole
T264,Escherichia-Shigella other
T181,Ruminococcaceae UCG-002
M26,dimethyltrisulfide
T127,Lachnospiraceae other
M23,pentanoic acid
M18,3-methylbutanoic acid
M17,butyric acid
T280,Akkermansia
T180,Ruminococcaceae other
M12,dimethyldisulfide
M38,phenol
T119,Lachnoclostridium
M32,benzaldehyde
M33,limonene
M5,ethyl acetate
T255,Sutterella
M74,benzenepropanoic acid
T77,Clostridium sensu stricto 1
T27,Bacteroides
M48,p-cresol
T41,Alistipes other
M66,1-hexadecene
M102,tetradecanoic acid
M46,4-methylbenzaldehyde
M56,octanoic acid
M43,linalol
M86,dodecanoic acid
M39,phenylacetadehyde
T160,Faecalibacterium
M73,decanoic acid
T193,Subdoligranulum
T135,Roseburia
T37,Prevotella 9
M34,hexanoic acid
T159,Eubacterium coprostanoligenes group
T40,Prevotellaceae NK3B31 group
T217,Dialister
T125,Lachnospiraceae NK4A136 group
T120,Lachnospira
T108,Eubacterium eligens group
1.8
1.6
1.4
1.2
1
0.8
0.6
0.4
0.2
0
-0.2
Inner squared distance

Suppl. Fig. S3. Richness in OTUs (A) and predicted KOs (B) in microbiota the cultures inoculated with the microbiota of subjects V1, V3, V4, V5, and V6, at 0, 6, and 12 h. * indicate statistical significant difference compared to 0 h (paired samples t-test, P < 0.05).

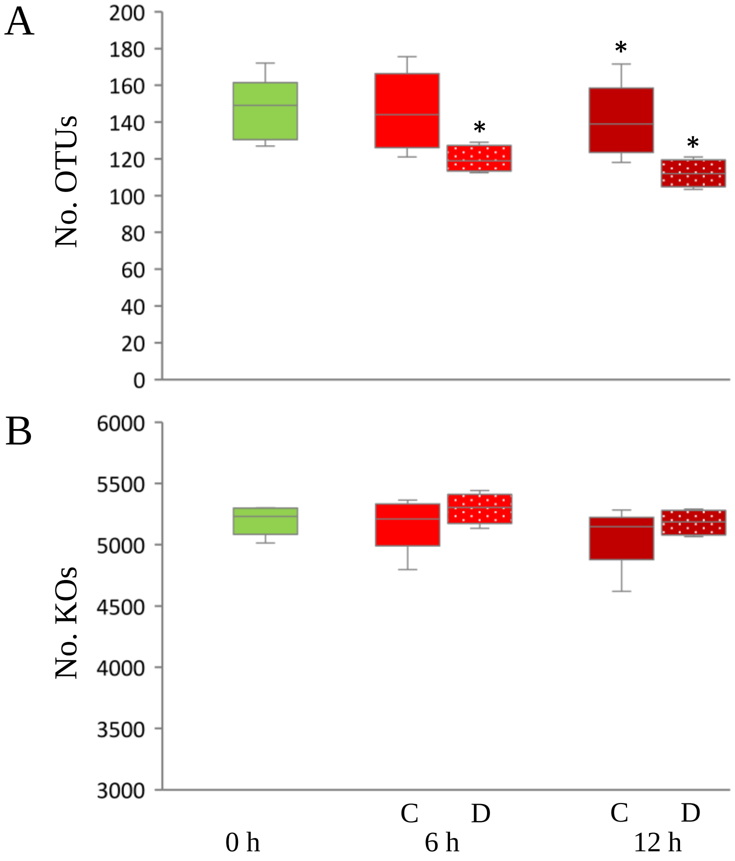

Suppl. Fig. S4. Predicted network of metabolic pathways. The KOs in the core metagenome that decreased (red), did not significantly change (yellow), or increased (green), and those, absent in the core metagenome that appeared during cultivation (blue) are reported. The image was produced with data obtained from KEGG annotation and visualized with iPath 3. https://pathways.embl.de/

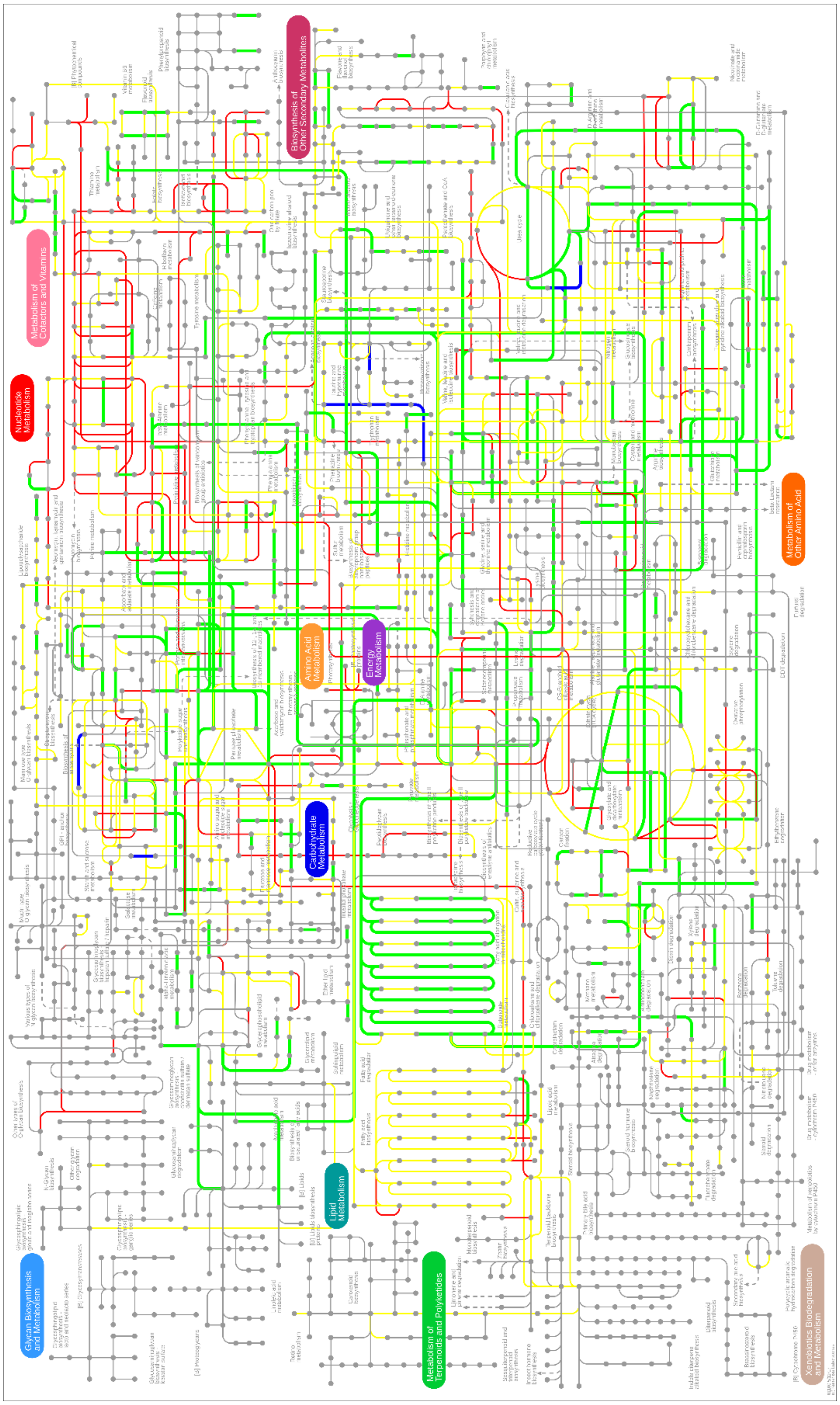

Suppl. Fig. S5. Predicted ABC (A) and PTS (B) transporters. The KOs in the core metagenome that decreased (red), did not significantly change (yellow), or increased (green) are reported. Annotation and figures obtained from KEGG.

A

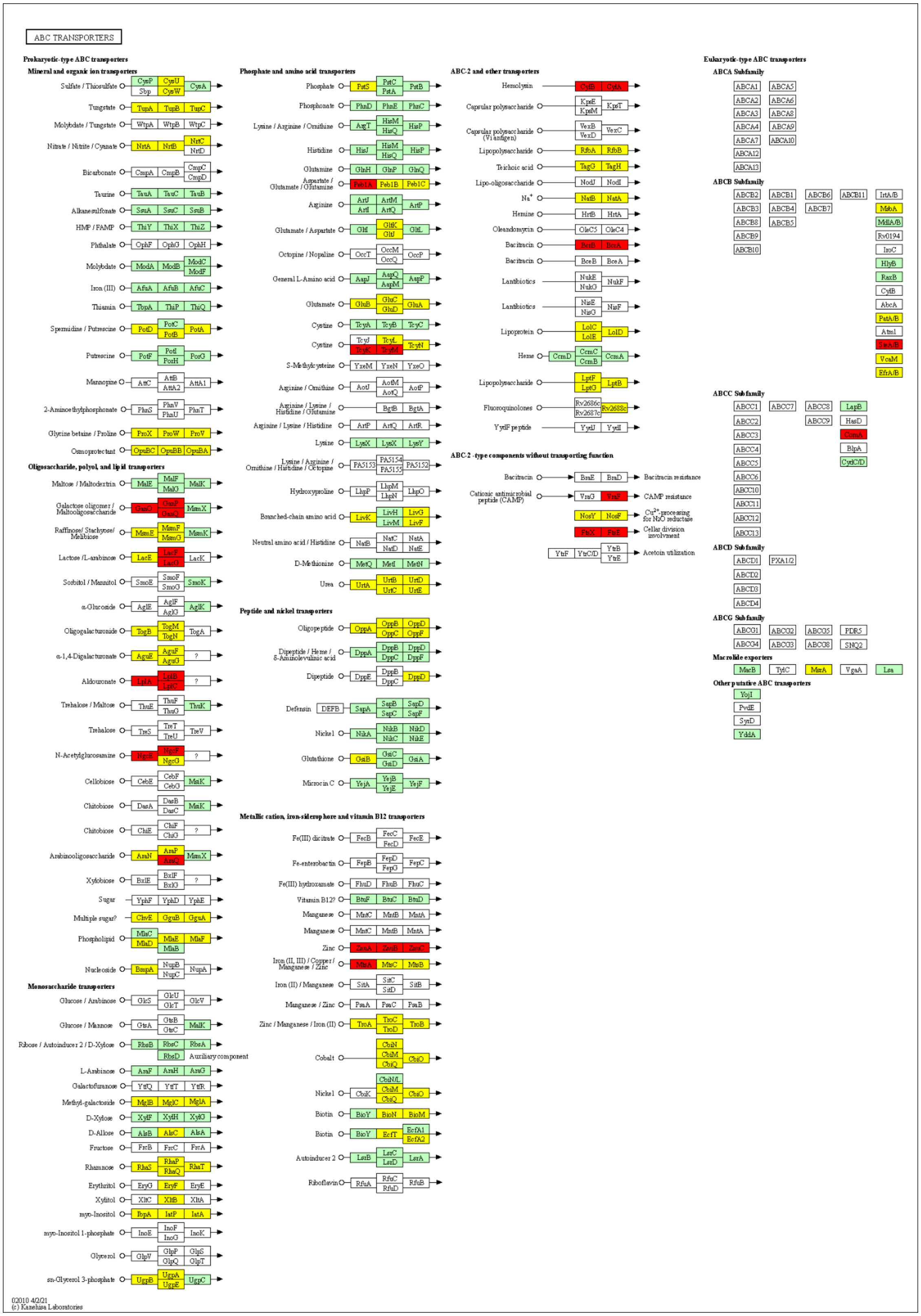

B

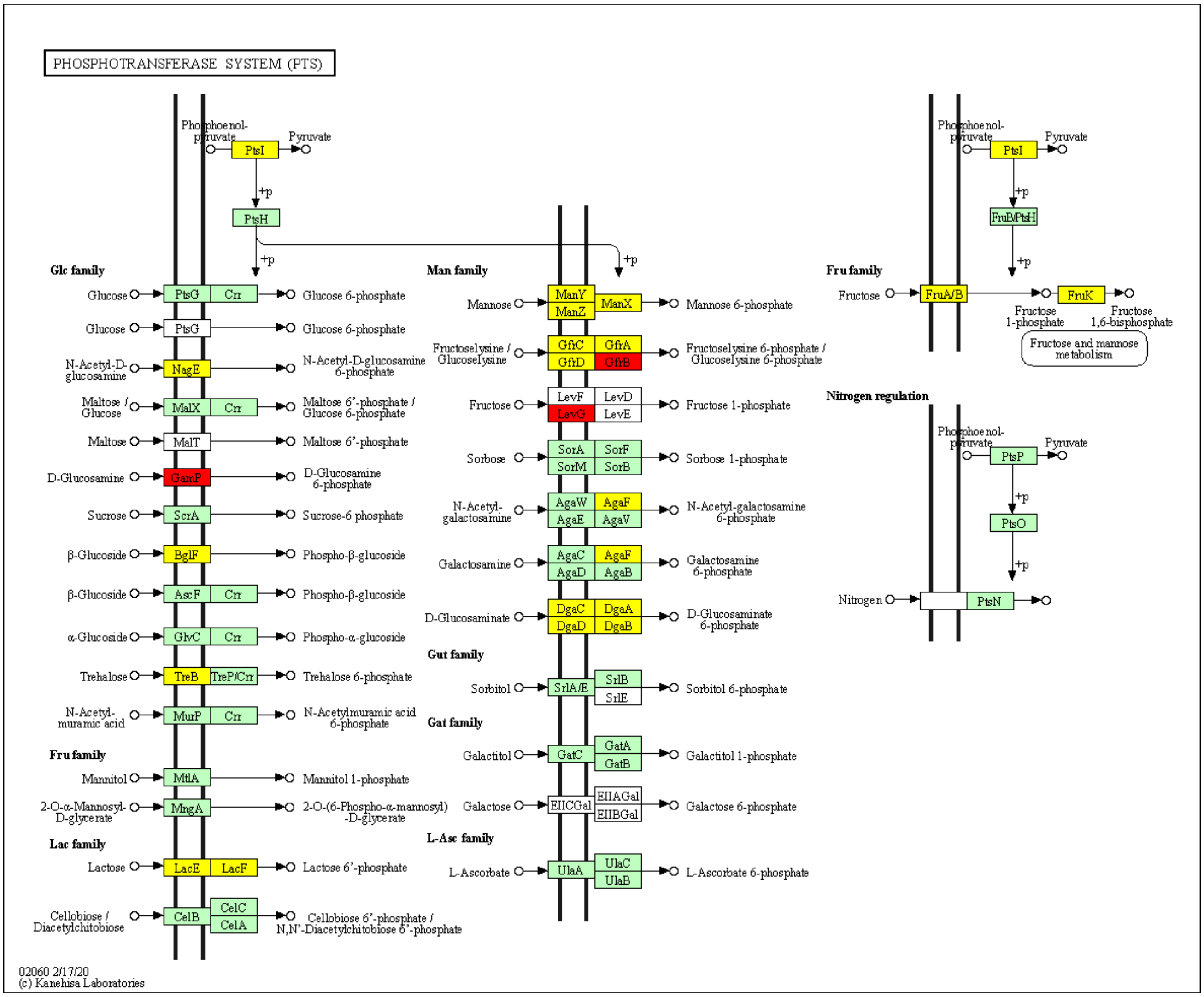
PHOSPHOTRANSFERASE SYSTEM (PTS)
Phosphoenol-pyruvate
PtsI
Pyruvate
+p
PtsH
+p
Glc family
Glucose
PtsG
Crr
Glucose 6-phosphate
Glucose
PtsG
Glucose 6-phosphate
N-Acetyl-D-glucosamine
NagE
N-Acetyl-D-glucosamine 6-phosphate
Maltose / Glucose
MalX
Crr
Maltose 6'-phosphate / Glucose 6-phosphate
Maltose
MalT
Maltose 6'-phosphate
D-Glucosamine
GamP
D-Glucosamine 6-phosphate
Sucrose
ScrA
Sucrose-6 phosphate
β-Glucoside
BglF
Phospho-β-glucoside
β-Glucoside
AscF
Crr
Phospho-β-glucoside
α-Glucoside
GlvC
Crr
Phospho-α-glucoside
Trehalose
TreB
TreP/Crr
Trehalose 6-phosphate
N-Acetyl-muramic acid
MurP
Crr
N-Acetylmuramic acid 6-phosphate
Fru family
Mannitol
MtlA
Mannitol 1-phosphate
2-O-α-Mannosyl-D-glycerate
MngA
2-O-(6-Phospho-α-mannosyl)-D-glycerate
Lac family
Lactose
LacE
LacF
Lactose 6'-phosphate
Cellobiose / Diacetylchitobiose
CelB
CelC
CelA
Cellobiose 6'-phosphate / N,N'-Diacetylchitobiose 6'-phosphate
Man family
Mannose
ManY
ManZ
ManX
Mannose 6-phosphate
Fructoselysine / Glucoselysine
GfrC
GfrD
GfrA
GfrB
Fructoselysine 6-phosphate / Glucoselysine 6-phosphate
Fructose
LevF
LevG
LevD
LevE
Fructose 1-phosphate
Sorbose
SorA
SorM
SorF
SorB
Sorbose 1-phosphate
N-Acetyl-galactosamine
AgaW
AgaE
AgaF
AgaV
N-Acetyl-galactosamine 6-phosphate
Galactosamine
AgaC
AgaD
AgaF
AgaB
Galactosamine 6-phosphate
D-Glucosaminate
DgaC
DgaD
DgaA
DgaB
D-Glucosaminate 6-phosphate
Gut family
Sorbitol
SrlA/E
SrlB
SrlE
Sorbitol 6-phosphate
Gat family
Galactitol
GatC
GatA
GatB
Galactitol 1-phosphate
Galactose
EIICGal
EIIAGal
EIIBGal
Galactose 6-phosphate
L-Asc family
L-Ascorbate
UlaA
UlaC
UlaB
L-Ascorbate 6-phosphate
Phosphoenol-pyruvate
PtsI
Pyruvate
+p
FruB/PtsH
+p
Fru family
Fructose
FruA/B
Fructose 1-phosphate
FruK
Fructose 1,6-bisphosphate
Fructose and mannose metabolism
Nitrogen regulation
Phosphoenol-pyruvate
PtsP
Pyruvate
+p
PtsO
+p
Nitrogen
PtsN
02060 2/17/20
(c) Kanehisa Laboratories
